# Effects of Age on Liver Enzyme Levels of Obese Men following Moderate-intensity Interval Training

**DOI:** 10.5005/jp-journals-10018-1184

**Published:** 2016-12-01

**Authors:** Valizadeh Rohollah, Karampour Sedigheh, Mahmoudi Yadollah, Khanmohammadi Rahmatollah, Mosavi Mojtaba

**Affiliations:** 1 Department of Physical Education, Omidiyeh Branch, Islamic Azad University, Omidiyeh, Islamic Republic of Iran; 2 Young Researchers and Elite Club, Omidiyeh Branch, Islamic Azad University, Omidiyeh, Islamic Republic of Iran; 3 Department of Physical Education Arkavaz Branch, Payamenour University, Arkavaz, Islamic Republic of Iran; 4 Department of Physical Education, Behbahan Branch Islamic Azad University, Behbahan, Islamic Republic of Iran

**Keywords:** Liver enzymes, Moderate-intensity interval training, Obese.

## Abstract

The present study aims to consider the effects of age on liver enzyme levels of obese men following 15 weeks of moderate-intensity interval training (MIIT). Ten obese middle-aged persons (aged 55.2 ± 4.31 years; height 171.4 ± 3.59 cm; weight 95.7 ± 14.61 kg; and body mass index [BMI] 32.53 ± 4.59 kg/m^2^) and 10 obese young adults (age 22.7 ± 1.25 years; height 177.4 ± 3.94 cm; weight 100.7 ± 8.04 kg; and BMI 32 ± 2.39 kg/m^2^) were enrolled in this study. Interval training was followed by 65 to 75% of VO_2_max, 1 set, 2 minutes and seven repetitions, 1 minute rest–relief interval with 50% of VO_2_max. Blood sampling was carried out in order to measure aspartate aminotransferase (AST), alanine aminotransferase (ALT), and alkaline phosphatase (ALP). All analyses were conducted by Statistical Package for the Social Sciences (SPSS) software (version 16 for windows). A descriptive statistics including mean and standard deviation was obtained for all parameters. Analysis of covariance (ANCOVA) and test of normality (Shapiro–Wilk test) were used. Paired sample *t*-test was used to compare the pretest and posttest data in each group. Comparison between middle-aged and young adults has shown that MIIT causes significant increase of ALT levels in young adults. There is no significant difference between middle-aged and young adults on other variables. Also paired sample *t*-test shows that ALP was decreased in both groups.

**How to cite this article:**

Rohollah V, Sedigheh K, Yadollah M, Rahmatollah K, Mojtaba M. Effects of Age on Liver Enzyme Levels of Obese Men following Moderate-intensity Interval Training. Euroasian J Hepato-Gastroenterol 2016;6(2):131-133.

## INTRODUCTION

The liver is the largest organ of the human body. It is located between the portal and the general circulation, between the organs of the gastrointestinal tract and the heart. The main and important role of the liver is to take up nutrients, in order to store them and also provide nutrients to the other organs. Almost all blood that enters the liver via the portal tract originates from the gastrointestinal tract as well as from the spleen, pancreas, and gallbladder. A second blood supply to the liver comes from the hepatic artery, branching directly from the celiac trunk and descending aorta.^1^ The most common liver enzymes are alanine aminotransferase (ALT) and aspartate aminotransferase (AST). These enzymes, the most reliable markers of liver injury, are secreted into the bloodstream when liver cells are injured. Some studies show that ALT and AST are significantly increased after exercise.^2^ The study has checked the influence of age on liver enzyme levels of obese men following 15 weeks of moderate-intensity interval training (MIIT).

## MATERIALS AND METHODS

This research has been formulated after accomplishing pretest and posttest assessment in two experimental groups. The participants gave written consent to the study (10 middle-aged and 10 young adults). The study protocol has been approved by the Research Ethics Committee of Islamic Azad University Omidiyeh Branch, Omidiyeh, Islamic Republic of Iran. Interval training was followed by 65 to 75% of VO_2_max, 1 set, 2 minutes and seven repetitions, 1 minute rest–relief interval with 50% of VO_2_max. Blood sampling has been done for the evaluation of ALT, AST, and alkaline phosphatase (ALP).

### Statistical Analysis

All analyses were conducted by Statistical Package for the Social Sciences (SPSS) software (version 16 for windows). A descriptive statistics including mean and standard deviation was obtained for all parameters. Analysis of covariance, paired sample *t*-test, and test of normality (Shapiro–Wilk test) were used.

## RESULTS

Table 1 shows all information about experimental groups.

**Table 1:** **Table 1:** Personal characteristics of young and middle-aged obese adults

*Variables*		*Groups*		*Number*		*Mean*		*Standard deviation*	
Age		Young		10		22.7		1.25167	
		Middle aged		10		55.2		4.31535	
Height		Young		10		177.4		3.94968	
		Middle aged		10		171.4		3.59629	
Weight		Young		10		100.7		8.04225	
		Middle aged		10		95.7		14.61392	
BMI		Young		10		32		2.39827	
		Middle aged		10		32.53		4.59696	

Analysis of covariance of liver enzymes in young and middle-aged obese people shows that there is no difference between AST and ALP. However, there was a significant difference between ALT of two groups (Table 2). Also, the results showed that young obese adults have higher ALT compared with middle-aged groups (Table 3).

**Table 2:** **Table 2:** Analysis of covariance of liver enzymes in young and middle-aged obese adults

*Variables*		*Source*		*Sum of square*		*df*		*Mean of square*		*F*		*Sig*	
AST		Covariance		1.681		1		1.681		0.068		0.797	
		Between group		1.095		1		1.095		0.044		0.836	
		Within group		420.319		17		24.725		–		–	
		Total		429.200		19		–		–		–	
ALT		Covariance		27.717		1		27.717		0.933		0.348	
		Between group		1023.697		1		1023.697		34.449		0.001	
		Within group		505.183		17		29.717		–		–	
		Total		1828.950		19		–		–		–	
ALP		Covariance		2187.315		1		2187.315		3.007		0.101	
		Between group		586.221		1		586.221		0.806		0.382	
		Within group		12365.985		17		727.411		–		–	
		Total		15309.750		19		–		–		–	

**Table 3:** **Table 3:** Paired sample *t*-test of young and middle-aged obese adults

*Variables*		*Groups*		*Mean ± SD of pretest*		*Mean ± SD of posttest*		*df*		*t*		*Sig*	
AST		Middle aged		19.30 ± 4.47		24.2 ± 6.69		9		–1.952		0.083	
		Young		29.7 ± 6.78		25.4 ± 1.42		9		2.076		0.068	
ALT		Middle aged		24.50 ± 7.56		26 ± 6.30		9		–0.487		0.638	
		Young		35.1 ± 7.40		42.1 ± 4.40		9		–2.077		0.068	
ALP		Middle aged		197.70 ± 19.27		174.10 ± 36.94		9		2.580		0.030	
		Young		256.60 ± 21.06		186.40 ± 15.88		9		8.358		0.001	

## DISCUSSION AND CONCLUSION

The results of the present study show that there is a significant difference between ALT of young and middle-aged obese adults. Also, the results show that MIIT causes more increase in ALT of young adults compared with middle-aged obese adults. However, more studies are needed to conclude that this type of training may be cautiously recommended for young obese adults and also led to liver cells damage in this population. Also, investigations have shown that 10 weeks resistance training with intensity of 50 to 90% of 1Maximal repetition (1MR) can lead to significant decrease in AST and ALT of middle-aged adults of fatty liver patients.^3^ So, it can be concluded that type of exercise and age are two important variables that should be considered before recommendation of exercise in obese subjects.
